# Plant traits associated with seed dispersal by ducks and geese in urban and natural habitats

**DOI:** 10.1002/ece3.10677

**Published:** 2023-11-22

**Authors:** Pál Tóth, Andy J. Green, David M. Wilkinson, Kane Brides, Ádám Lovas‐Kiss

**Affiliations:** ^1^ Hortobágyi National Park Directorate Debrecen Hungary; ^2^ University of Debrecen Pál Juhász‐Nagy Doctoral School Debrecen Hungary; ^3^ HUN‐REN, Centre for Ecological Research, IAE, Wetland Ecology Research Group Debrecen Hungary; ^4^ Department of Conservation Biology and Global Change Estación Biológica de Doñana, EBD‐CSIC Sevilla Spain; ^5^ School of Life and Environmental Sciences University of Lincoln Lincoln UK; ^6^ School of Biological and Environmental Sciences Liverpool John Moores University Liverpool UK; ^7^ Wildfowl & Wetlands Trust (WWT) Slimbridge UK; ^8^ One Health Institute, Faculty of Health Sciences University of Debrecen Debrecen Hungary; ^9^ HUN‐REN‐DE Conservation Biology Research Group Debrecen Hungary

**Keywords:** alien species, Anatidae, Canada goose, dispersal, endozoochory, mallard

## Abstract

Ducks and geese are little studied dispersal vectors for plants lacking a fleshy fruit, and our understanding of the traits associated with these plants is limited. We analyzed 507 faecal samples of mallard (*Anas platyrhynchos*) and Canada goose (*Branta canadensis*) from 18 natural and urban wetlands in England, where they are the dominant resident waterfowl. We recovered 930 plant diaspores from 39 taxa representing 18 families, including 28 terrestrial and five aquatic species and four aliens. Mallards had more seeds and seed species per sample than geese, more seeds from barochory and hydrochory syndromes, and seeds that on average were larger and from plants with greater moisture requirements (i.e., more aquatic). Mallards dispersed more plant species than geese in natural habitats. Plant communities and traits dispersed were different between urban (e.g., more achenes) and natural (e.g., more capsules) habitats. Waterfowl can readily spread alien species from urban into natural environments but also allow native terrestrial and aquatic plants to disperse in response to climate heating or other global change. Throughout the temperate regions of the Northern Hemisphere, the mallard is accompanied by a goose (either the Canada goose or the greylag goose) as the most abundant waterfowl in urbanized areas. This combination provides a previously overlooked seed dispersal service for plants with diverse traits.

## Graphical abstract

Ducks and geese are understudied dispersal vectors for plants lacking fleshy fruit, and our understanding of the traits associated with these plants is very limited. We analyzed 507 faecal samples of mallard (*Anas platyrhynchos*) and Canada goose (*Branta canadensis*) from 18 natural and urban wetlands in England, where they are the dominant resident waterfowl. Waterfowl can readily spread alien species from urban into natural environments but also allow native terrestrial and aquatic plants to disperse in response to climate heating or other global change.

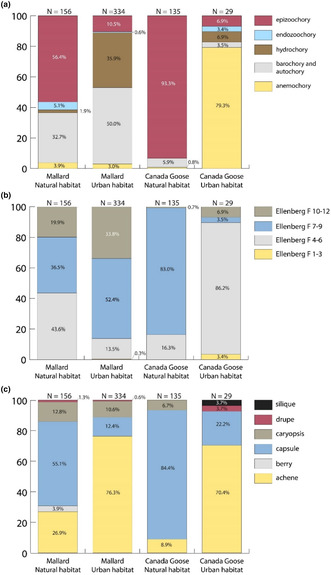

## 1 INTRODUCTION

Plant dispersal is strongly affected by human activities, both directly through movements of plants by humans and indirectly through modification of habitats and impacts on plant vectors such as birds and mammals (Daru et al., 2021; Emer et al., 2019). Research into plant dispersal via animal vectors (zoochory) has focused principally on dispersal of vascular plants by fruit‐eating and scatter‐hoarding vertebrates (Forget et al., 2011; Pesendorfer et al., 2016). However, non‐frugivorous birds such as waterfowl (e.g., ducks, geese, and swans) are now known to disperse a broad range of angiosperms in a manner not predicted by popular morphological dispersal syndromes (Green et al., 2022; Urgyán et al., 2023). Therefore, other traits should be explored to characterize the plants dispersed by these vectors.

Ducks can be excellent vectors of plants in and around isolated ponds, lakes, and other wetlands that lack hydrological connections, and they disperse more seeds by endozoochory (i.e., in the digestive tract) than by epizoochory (i.e., on feathers or skin; Brochet et al., 2010; Green et al., 2022, 2023). Mallards are one of the world's most abundant dabbling duck species (BirdLife International, 2023). As opportunistic habitat generalists, they ingest and disperse an abundance of seeds from a wide range of plant species (Kleyheeg et al., 2017; Soons et al., 2016; Urgyán et al., 2023). Recent studies demonstrate that geese are also important seed vectors in Europe (Almeida et al., 2022; Hattermann et al., 2019; Lovas‐Kiss et al., 2023; Navarro‐Ramos et al., 2024), but there are no studies comparing plants dispersed by ducks and geese in the same habitats, or their trait composition.

Waterfowl provide an important ecosystem service through seed dispersal of native plants (Green et al., 2016; Green & Elmberg, 2014), although they may also spread alien species (Green, 2016) and agricultural weeds (Navarro‐Ramos et al., 2024). Some waterfowl are themselves alien species, and introductions of alien birds can have major impacts on ecosystems (Evans et al., 2021; Mooney & Cleland, 2001). On the other hand, alien birds can play a positive role in seed dispersal, may partially compensate for the loss of native birds (Kawakami et al., 2009; La Rosa et al., 1985; Martin‐Albarracin et al., 2018), and can quickly integrate into plant–vector interaction networks (Vizentin‐Bugoni et al., 2019). Non‐native Canada geese (*Branta canadensis*) are among the commonest breeding waterbirds in the UK (Frost et al., 2019) and can have strong negative impacts through fouling of public urban spaces, crop consumption, and as a risk to aviation safety (Evans et al., 2020). However, cost–benefit analyses of the impact of this species (Reyns et al., 2018) do not consider their role in seed dispersal.

With the ongoing loss of natural wetlands across the globe, artificial wetlands are becoming increasingly important as habitat for waterbirds (Ma et al., 2010; Murray et al., 2013; Navedo et al., 2012), although they are generally not the functional equivalents of natural wetlands (Almeida et al., 2020; Campbell et al., 2002) and typically support different plant communities with more ruderal and alien species. Waterbird populations are often dependent on a combination of natural and artificial wetlands, and individuals regularly move between them, facilitating seed dispersal (Almeida et al., 2020). Contact with waterfowl is appreciated by humans, and some species (e.g., mallards and Canada geese) are common in urban habitats. However, waterfowl endozoochory in urban environments has not previously been studied. Studies have shown that urbanization has a homogenization effect on frugivore seed dispersal networks (Schneiberg et al., 2020), although frugivore networks can be temporally complex in urban parks (Cruz et al., 2013).

According to widespread assumptions, certain plant traits (e.g., plumes, hairs, wings, floating devices, nutritive tissue, sticky surfaces, hooks on seeds) are putative adaptations for dispersal that can be used to predict the dispersal vector (Traveset et al., 2014; Van der Pijl, 1982). Hence, each angiosperm species has been assigned to different morphological dispersal syndromes (see definitions in Table S3) in databases such as Baseflor (Julve, 1998). Plant functional traits (including dispersal syndromes) have been widely applied to study ecological processes (Funk et al., 2017). However, for waterbirds, ungulates and many other vectors, dispersal syndromes do not mirror dispersal mechanisms (Green et al., 2022), and a new approach is required. Syndromes may still be informative since they are based on specific traits, e.g., buoyant structures diagnostic for a “hydrochory syndrome” might increase availability to surface‐feeding waterbirds. Diet studies suggest that other plant traits such as moisture requirements and seed size help predict waterfowl endozoochory (Almeida et al., 2022; Soons et al., 2016).

In this study, we used faecal analysis to compare seed dispersal by the two most abundant resident species of waterfowl in England, the native mallard and the non‐native Canada goose, using functional traits to characterize the plants dispersed in urban or natural settings. Mallards are the best‐studied seed disperser among the Anatidae (Lovas‐Kiss et al., 2018; Soons et al., 2016; Urgyán et al., 2023), but previous studies in the UK are limited to studies of upper gut contents (Sebastián‐González et al., 2020). Canada geese are known to disperse plants in their native range in Greenland and North America (Green et al., 2016, 2018), but their role as plant vectors has not previously been studied in the introduced range. Our initial hypotheses were as follows:

1. Mallards (dabbling ducks that are largely granivorous and feed on water) and Canada geese (larger herbivores grazing on land) would disperse plant species with different functional traits in a given site, and numbers and species diversity of seeds would also differ. Previous studies suggest that geese may ingest more seeds of terrestrial plants, and those with fleshy fruit, than dabbling ducks such as mallards (Almeida et al., 2022; Green et al., 2016, 2018), but they have never been studied together in the same area. Mallards may be expected to feed more on large, obvious seeds, whereas geese may ingest more small seeds while grazing on foliage.
2. In urban habitats, waterfowl would disperse different plant species with different traits, including ornamental aliens planted in gardens and parks. Seed production of many species in urban parks may be limited through intense management, e.g., mowing and use of herbicides.
3. Endozoochory rates depend on plant phenology. We considered seasonal trends in endozoochory rates and compared them between species or habitats. The extent to which endozoochory is coupled with seed production is an important determinant of the direction of plant dispersal (González‐Varo et al., 2021; Urgyán et al., 2023). In the UK, seed production is concentrated in late summer (Preston et al., 2002).

## 2 MATERIALS AND METHODS

### 2.1 Study species

The mallard (c.1 kg, Kear, 2005) is the most abundant breeding duck species in the UK (59,000–140,000 individuals) and the rest of Europe (Bird Life International, 2023; Hagemeijer & Blair, 1997). The UK population contains a mixture of sedentary to fully migratory birds, with ringing recoveries away from the UK of up to 2827 km (Robinson et al., 2021; Wernham et al., 2002). Inhabiting almost every wetland type, it feeds mainly on seeds, green plant material and invertebrates taken in shallow water and on land (Dessborn et al., 2011; Kear, 2005).

The Canada goose (3–5 kg) is native to North America and was first introduced to the UK in 1665. It became established in the wild in the late 19th century, and it is now the non‐native bird with the second highest biomass in the UK (Yalden & Albarella, 2009). The UK population is resident, although movements exceeding 150 km are commonplace, especially during moult migrations in May/June and later in August (Brides et al., 2023), and some individuals have moved to continental Europe and even the USA (Robinson et al., 2021; Wernham et al., 2002). The Canada goose prefers grazing in open, grassy habitats in fields, parks or around wetland edges (Jansson et al., 2008). Although it is territorial during the breeding season, it typically co‐occurs with mallards.

Both species regularly undergo movements between different wetlands in a given region (Wernham et al., 2002) and are likely to connect urban and natural habitats. Ringing recoveries confirm movements of individuals between different study sites (Brides et al., 2023).

### 2.2 Study areas and sample processing

**FIGURE 1:**
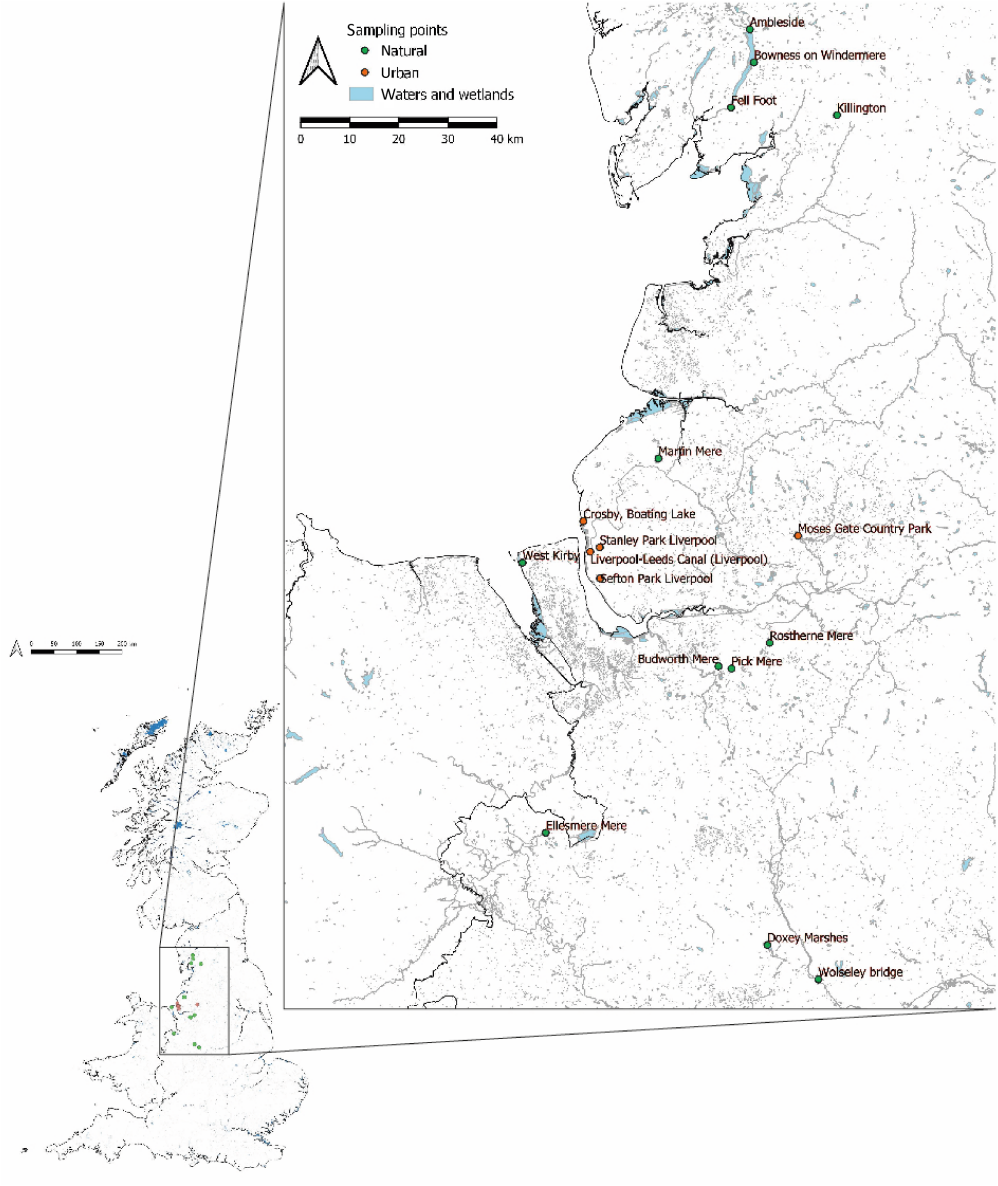
Study locations.

Faecal samples (Mallard *n* = 257, Canada geese *n* = 250) were collected in north‐west England (Figure 1, Table S1) from 18 sites, often sampling both species at the same site. Our observations suggested the sampled birds had been feeding in and around the collection sites. We sampled in urban areas (including parks and canals) as well as natural lakes and other natural wetlands in spring (*n* = 6 days), summer (*n* = 13 days) and autumn (*n* = 6 days) in 2016 (*n* = 21 days) and 2019 (*n* = 4 days). Although naturalness is a continuum, England does not contain habitats untouched by human activities, and many of our sites might be considered as “semi‐natural”. We divided our sites a priori into “urban” (*n* = 6) and “natural” (*n* = 12) based on the extent of surrounding urban land‐use, and presence of artificial, paved shorelines that are common in urban parks and canals (Tables S1 and S4).

When sampling, we moved toward resting individuals or flocks and sampled fresh faeces after the birds had moved away, leaving at least 1 m between samples to minimize the chances of repeated sampling of the same individual. We never collected a higher number of samples than there were individual birds. Samples were checked in the field for contamination with soil or diaspores that might have adhered after egestion, lifted off the substrate with a clean penknife, placed in zip‐lock bags, and refrigerated. Later, the mass of each sample was measured before separation of plant diaspores, by washing with deionized water on a 100‐μm sieve. Only intact angiosperm diaspores (‘seeds’ from here on, but including vegetative propagules of duckweed *Lemna*) were collected. Identification was made to species level where possible, under a stereomicroscope, by comparing the morphology (shape, size, colour and seed coat pattern) of seeds with available literature (Bojnanský & Fargašová, 2007; Cappers et al., 2012; Preston et al., 2002). For each species identified, we extracted functional traits including morphological dispersal syndrome (Table S3) and fruit types from the Baseflor database (Julve, 1998). We obtained seed length and width from Cappers et al. (2012). As an indication of moisture requirements, we extracted Ellenberg *F* indicators for each species from Julve (1998), Hill et al. (1999) and Domina et al., 2018. We considered plants with values of 1–4 to be dry soil terrestrial, 5–8 to be moist soil terrestrial and 9–12 to be aquatic.

In order to demonstrate dispersal, we tested whether intact seeds were viable. For germinability or growth tests, we placed seeds in Petri dishes filled with bacteriological agar or cell culture plates filled with distilled water (for submerged plants and *Lemna* plantlets) placed on the laboratory windowsill. Germination tests were run for up to two months.

### 2.3 Statistical analysis

We used a forward model selection, adding in each step an independent variable to the models. The final models were selected based on the AIC and BIC values. Generalized linear mixed model with binomial error distribution was used to investigate differences between bird species and habitat types in presence (1) or absence (0) of seeds in each sample (Bates et al., 2015). Predictors in the model were bird species and habitat type (urban or natural) as fixed factors, sample mass as a continuous variable, and collection date as a random factor. The GlmmTMB (Brooks et al., 2017) package were used to build negative binomial distribution models to test for differences between bird species or habitat type in the total number of seeds per sample. This model was formulated as above, using bird species, habitat and sample mass as predictors, while controlling for the collection date with a random factor. When testing the differences in the total number of species per sample between bird species and habitats, we again included bird species, habitat type and sample mass as predictors, while controlling for the collection date as random factor.

We compared the frequencies of Ellenberg *F* values, length and width of seeds recovered from samples from different bird species or habitats, using only data from seeds identified at the species level and applying linear mixed models (lmer, ‘lme4’ package, Bates et al., 2015) with Gaussian error distribution. For each sample, we first calculated the weighted mean for seed length, width and Ellenberg *F*, and then used this as the dependent variable, with bird species and habitat types as fixed predictors and also collection date as random factor.

To visualize differences in the dispersed communities between waterbird species and habitat types, we used Bayesian ordination and regression analysis (boral) models using the “boral” package in R, with default parameters for controlling the Markov chain Monte Carlo sampling (Hui, 2016, 2018) using all the samples with raw abundance data. In the ordination, we used negative binomial error distribution due to the dominance of samples without any seeds, and the samples ID‐s were also included so the model is based on the composition of species. Bird species and habitat types were included as predictors. To identify plant taxa which drive differences in species composition among the different bird species and habitats, we used generalized linear models for multivariate abundance data (manyglm), with negative binomial error distribution and log link, and an unknown overdispersion parameter, using the “mvabund” package in R (Wang et al., 2012).

To identify seasonal trends from April to October in the total number of dispersed seeds per sample, for each bird species and habitat, we chose a non‐linear regression method, i.e., the function ‘loess’ for local polynomial regression fitting from the “stats” package (R Core Team, 2021, using 75% span). The total number of seeds (log transformed) was the dependent variable, and calendar day was the predictor (with 1 as 1st January). Rarefaction analysis was used to compare species richness found in samples collected from different species and habitats using the “iNEXT” (Chao et al., 2014) package.

All statistical analyses were performed in R software (RStudio 2021.09.2 Build 382; RStudio, PBC, 2021).

## 3 RESULTS

From all collected samples from any bird species, habitat or season (*n* = 507), we recovered 930 propagules, representing 39 plant taxa from 18 families (Table 1), including 5 aquatic species (two submerged, one floating, two emergent), 28 terrestrial species and 6 taxa identified to family level. Four species were aliens (Table 1). Moisture (Ellenberg *F*) values ranged from 3 to 12, i.e., from dry to fully aquatic habitats. Only five species have a fleshy fruit and are thus considered to have an “endozoochory syndrome”, and 21 have abiotic dispersal syndromes (Table 1).

**TABLE 1:** Total number of propagules (TP), number of samples (NS), maximum number of propagules in one sample (Tmax) and number of germinated propagules (GP) for each plant taxon, together with functional traits (dispersal syndrome, fruit type, Ellenberg *F* value, seed length, and width).

Propagule		Dispersal syndrome^a^	Fruit type^a^	Ellenberg *F* ^b^	Length (mm)	Width (mm)	Mallard (*n* = 257)								Canada goose (*n* = 250)							
Family	Species						Natural (*n* = 129)				Urban (*n* = 128)				Natural (*n* = 185)				Urban (*n* = 65)			
							TP	NS	Tmax	GP	TP	NS	Tmax	GP	TP	NS	Tmax	GP	TP	NS	Tmax	GP
Adoxaceae	*Sambucus nigra*	Endozoochory	Drupe	5	3.41	1.60	1	1	1	0	–	–	–	–	–	–	–	–	–	–	–	–
Anacardiaceae	***Rhus typhina*** ^c^	Endozoochory	Drupe	3	3.57	2.75	–	–	–	–	1	1	1	0	–	–	–	–	–	–	–	–
Apiaceae	–						–	–	–	–	2	1	2	0	–	–	–	–	–	–	–	–
Araceae	*Vegetative **Lemna minuta*** ^c^	Hydrochory	–	11	–	–	–	–	–	–	51	9	19	0	–	–	–	–	2	1	2	0
Asteraceae	*Bellis perennis*	Barochory	Achene	5	2.99	1.49	–	–	–	–	–	–	–	–	1	1	1	0	–	–	–	–
	*Cirsium vulgare*	Anemochory	Achene	5	4.36	1.69	1	1	1	0	–	–	–	–	–	–	–	–	–	–	–	–
	*Helianthus annuus*	Barochory	Achene	6	9.81	6.40	–			–	–	–	–	–	1	1	1	0	–	–	–	–
Betulaceae	*Betula pendula*	Anemochory	Achene	5	6.44	4.09	3	3	1	0	10	7	2	0	–	–	–	–	19	1	19	0
Brassicaceae	***Lepidium dydimus*** ^c^	Anemochory	Silique	3	1.06	0.76	–	–	–	–	–	–	–	–	–	–	–	–	1	1	1	0
Caryophyllaceae	*Sagina apetala*	Anemochory	Capsule	4	0.33	0.28	–	–	–	–	–	–	–	–	1	1	1	0	3	3	1	1
	*Sagina procumbens*	Barochory	Capsule	6	0.38	0.31	–	–	–	–	–	–	–	–	–	–	–	–	1	1	1	0
Cucurbitaceae	*Bryonia alba*	Endozoochory	Berry	5	4.08	3.27	6	1	6	1	–	–	–	–	–	–	–	–	–	–	–	–
Cyperaceae	*Carex pendula*	Barochory	Achene	8	1.92	1.06	–	–	–	–	134	6	120	26	–	–	–	–	–	–	–	–
	*Cyperus* sp.						1	1	1	0	–	–	–	–	–	–	–	–	–	–	–	–
Geraniaceae	*Geranium robertianum*	Autochory	Capsule	6	2.05	1.14	1	1	1	0	–	–	–	–	–	–	–	–	–	–	–	–
Juncaceae	*Juncus bufonius*	Epizoochory	Capsule	7	0.44	0.25	48	7	30	4	34	1	34	2	112	7	67	38	1	1	1	0
	*Juncus compressus*	Epizoochory	Capsule	8	0.49	0.24	6	1	6	2	–	–	–	–	–	–	–	–	–	–	–	–
	*Juncus effusus*	Epizoochory	Capsule	7	0.56	0.26	1	1	1	0	–	–	–	–	–	–	–	–	–	–	–	–
	*Juncus* sp.						225	4	123	7	5	1	5	0	–	–	–	–	15	2	11	0
Plantaginaceae	*Callitriche* sp.						1	1	1	0	–	–	–	–	–	–	–	–	–	–	–	–
	***Kickxia spuria*** ^d^	Epizoochory	Capsule	4	1.37	0.78	–	–	–	–	1	1	1	0	–	–	–	–	–	–	–	–
	*Plantago media*	Barochory	Achene	4	2.62	1.19	–	–	–	–	3	1	3	0	–	–	–	–	–	–	–	–
	*Veronica anagallis*‐*aquatica*	Barochory	Capsule	10	0.64	0.45	23	5	10	5	–	–	–	–	1	1	1	0	–	–	–	–
	*Veronica beccabunga*	Barochory	Capsule	10	0.66	0.56	7	1	7	6	–	–	–	–	–	–	–	–	–	–	–	–
	*Veronica montana*	Epizoochory	Capsule	6	2.19	1.81	–	–	–	–	–	–	–	–	–	–	–	–	1	1	1	0
Poaceae	*Agrostis stolonifera*	Barochory	Caryopsis	6	1.95	0.46	4	3	2	0	25	4	13	0	3	1	3	0	–	–	–	–
	*Alopecurus pratensis*	Barochory	Caryopsis	5	2.45	1.12	1	1	1	1	–	–	–	–	–	–	–	–	–	–	–	–
	*Anthoxanthum odoratum*	Epizoochory	Caryopsis	6	1.68	0.72	–	–	–	–	–	–	–	–	4	2	2	1	–	–	–	–
	*Lolium perenne*	Barochory	Caryopsis	5	3.72	1.15	–	–	–	–	5	1	5	1	2	2	1	0	–	–	–	–
	*Poa annua*	Barochory	Caryopsis	5	1.59	0.67	15	3	10	4	–	–	–	–	–	–	–	–	–	–	–	–
	–						20	6	9	0	1	1	1	0	3	3	1	1	–	–	–	–
Polygonaceae	*Rumex acetosa*	Anemochory	Achene	5	2.08	1.22	2	1	2	0	–	–	–	–	–	–	–	–	–	–	–	–
Potamogetonaceae	*Potamogeton pectinatus*	Hydrochory	Achene	12	3.27	2.48	–	–	–	–	61	7	16	10	–	–	–	–	–	–	–	–
	*Potamogeton pusillus*	Hydrochory	Achene	12	2.23	1.36	1	1	1	0	1	1	1	1	–	–	–	–	–	–	–	–
Ranunculaceae	*Ranunculus acris*	Epizoochory	Achene	6	3.56	2.32	33	3	20	0	–	–	–	–	10	3	5	3	–	–	–	–
	*Ranunculus sceleratus*	Hydrochory	Achene	8	1.28	0.95	2	1	2	0	7	2	5	–	–	–	–	–	–	–	–	–
Rosaceae	*Rubus fruticosus agg*.	Endozoochory	Drupe	6	3.08	2.02	1	1	1	0	–	–	–	–	–	–	–	–	–	–	–	–
	*Rubus idaeus*	Endozoochory	Drupe	5	2.78	1.62	–	–	–	–	1	1	1	0	–	–	–	–	1	1	1	0
	*Rubus* sp. *small*						3	2	2	0	–	–	–	–	–	–	–	–	–	–	–	–
Total							406			30	342			40	138			43	44			1

*Note*: Alien species are in bold.

^a^ From BASEFLOR database (Julve, 1998).

^b^ From ECOFACT research (Domina et al., 2018; Hill et al., 1999).

^c^ Neophyte.

^d^ Archaeophyte.

### 3.1 Comparing mallards and Canada geese as plant vectors

Mean sample mass was 3.23 ± 0.11 g (± SE) for mallard (*n* = 257) and 5.16 ± 0.26 g for Canada goose (*n* = 250). Mallard samples contained 697 intact seeds (from 25 plant species and 6 families) and 51 vegetative propagules, compared to 328 seeds (from 14 plant species and 2 families) and 2 vegetative propagules from geese. Of mallard samples, 26.1% (*n* = 67) had at least one intact propagule, compared to 8.4% (*n* = 21) of goose samples. In GLMMs the differences between bird species in seed prevalence (*z*‐value: 1.908, *p* = .056) was marginally significant, and mallards had a significantly higher number of plant species per sample (*z*‐value: 2.266, *p* = .024) and seeds per sample (*z*‐value: 3.689, *p* <.001). The proportion of seeds from alien species (Table 1) was higher for mallards (5.70%, *n* = 53) than for geese (1.2%, *n* = 3). The number of dispersed seeds per sample showed evidence of seasonal patterns for both mallards and geese. Although endozoochory was recorded from May to October, there was an increase in the number of seeds per sample in the second half of the year (Figure S1).

**FIGURE 2:**
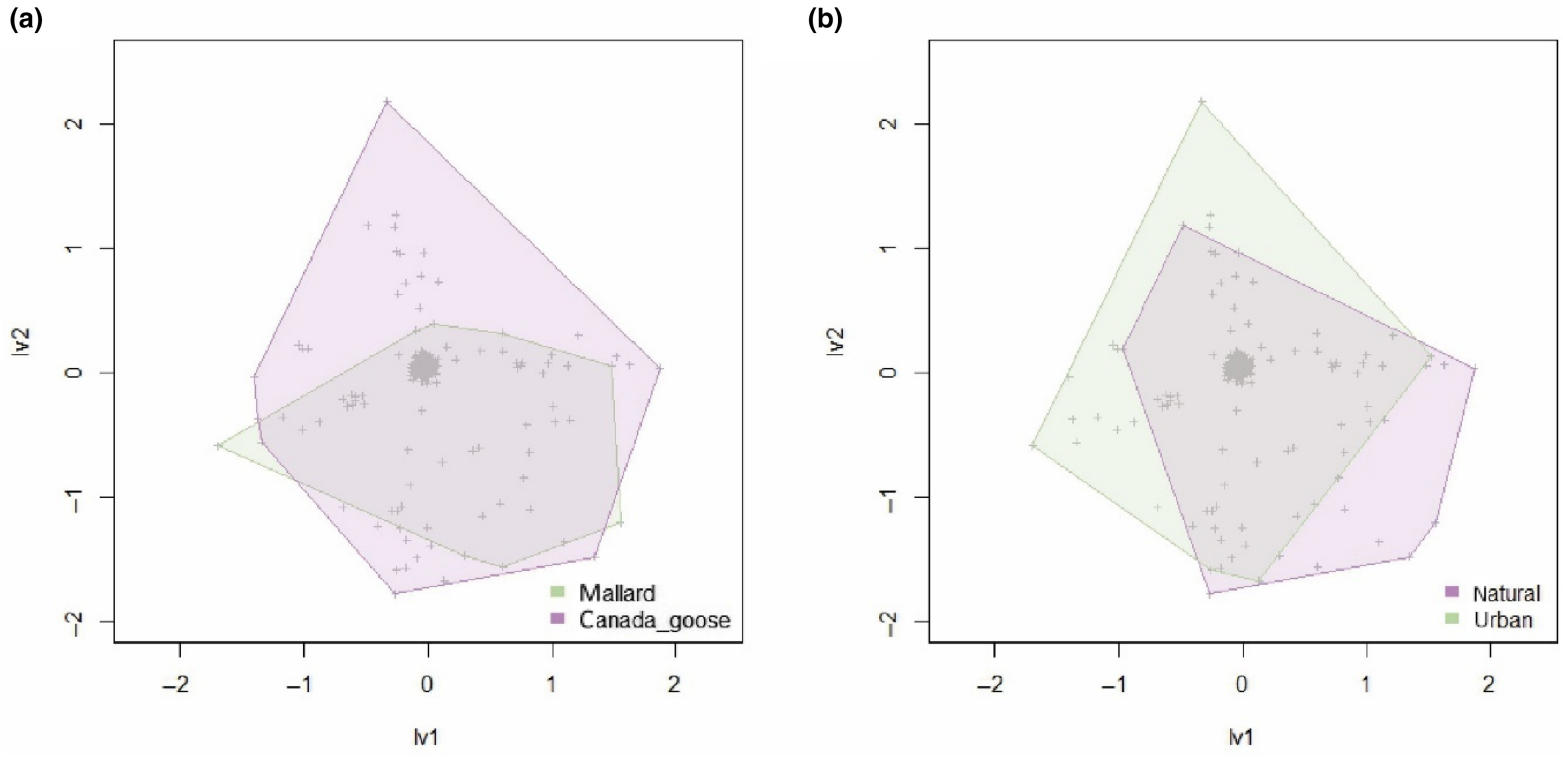
Ordination of latent variable models showing the differences between plant communities dispersed by mallards and Canada geese (a), and between plant communities dispersed by mallards and geese (combined) in different habitats (b). Axes represent latent variables 1 and 2. Each dot represents a sample (samples without a propagule are not excluded).

**FIGURE 3:**
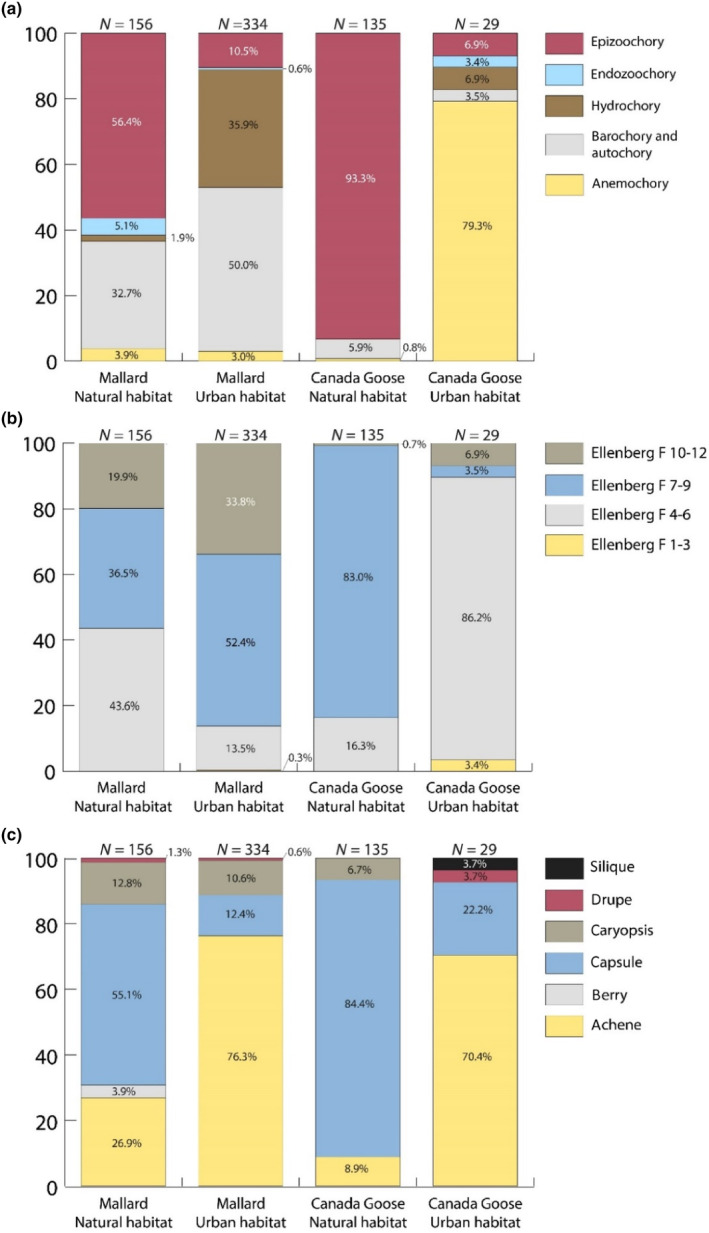
Trait frequency histograms for (a) dispersal syndromes, (b) Ellenberg *F* values, and (c) fruit types of seeds carried by mallards and Canada geese in different habitats. Sample sizes are given above each bar.

The plant communities dispersed by mallards and geese differed significantly (manyglm, *p* = .001, Figure 2). Fennel pondweed *Potamogeton pectinatus* was only recorded in mallards and made a significant contribution to the difference in community composition (*p* = .041). Seeds dispersed by mallards (median of the weighted means: 7.14) had significantly higher moisture (Ellenberg *F*) requirements than those dispersed by geese (median of the weighted mean: 6) (*t*‐value = 2.125, *p* = .037). Mallards dispersed seeds from aquatic and terrestrial plants in similar proportions, while geese dispersed mainly seeds of terrestrial plants (Figure 3b). No seeds of open‐water plant species (*F* = 12) were recorded in geese. Mallard samples also contained significantly longer (*t*‐value = 2.947, *p* = .005) and marginally significantly wider (*t* = 1.970, *p* = .053) seeds than geese samples (Figure S4).

Plant species with a fleshy fruit were better represented in mallards (five species, 2.0% of seeds) than in geese (one species, 0.6% of seeds). The proportion of fruit types in dispersed seeds also varied (Figure 3c), with mallards dispersing mainly achenes (76.3% of seeds), and geese mainly capsules (74.1%).

Seeds from 12 taxa dispersed by mallards (*n* = 70 seeds) and 6 taxa dispersed by geese (*n* = 44 seeds) were germinated (Table 1). Despite an initial healthy appearance, none of the least duckweed (*Lemna minuta*) plantlets recovered from feces (Table 1) grew in the lab.

### 3.2 Comparing endozoochory in natural and urban habitats

Rarefaction analysis showed that in natural environments, mallards dispersed significantly more plant species, while in urban environments there was no difference between geese and mallards (Figure S3). Overall, 17 plant species (4 families) were dispersed in urban habitats, compared to 23 plant species (5 families) in natural habitats.

In GLMMs, we found no significant differences between habitat types in the proportion of faecal samples containing at least one seed, species richness per sample, or seed abundance per sample (Table S2). There was evidence of seasonality in both habitat types, with more seeds dispersed per sample in the second half of the year (Figure S2).

Significantly different plant communities (*p* = .001, Figure 2) were dispersed in urban and natural habitats. Four species contributed significantly to these differences and were more abundant in urban habitats: *Lemna minuta* (*p* = .001), *P. pectinatus* (*p* = .002), pendulous sedge *Carex pendula* (*p* = .008) and silver birch *Betula pendula* (*p* = .034). Furthermore, all alien seeds were from urban environments. There was no significant difference in moisture requirement (Figure 3b), length or width of seeds between habitat types.

Dispersal syndromes and fruit types differed between habitat types (Figure 3). In natural habitats, epizoochory (mallard‐56% of seeds; geese‐93%) and barochory/autochory (mallard‐33%; geese‐6%) are the most characteristic syndromes, while in urban habitats barochory/autochory (50%) and hydrochory (36%) were the most important for mallards, and anemochory (79%) for geese (Figure 3a). In natural habitats, capsules were dominant for both bird species (mallards 55% of seeds; geese 84%), whereas achenes (mallards 76%; geese 70%) dominated in urban areas (Figure 3c).

## 4 DISCUSSION

Our detailed comparison of endozoochory by one native and another non‐native waterfowl in natural and urban environments in the UK identified important differences in their roles as seed vectors, and in the traits of plants they disperse. Mallards dispersed more seeds and a more diverse plant community than Canada geese (Figure S3). They dispersed more aquatic species, and species with larger seeds. At urban sites, both waterfowl were more likely to disperse plant species with high moisture requirements, as well as alien species. Dispersal syndromes differed between bird species and habitats, suggesting their diagnostic traits (Table S3) provide useful information about the likelihood of endozoochory by waterfowl.

### 4.1 Overlooked vectors of native plants in the UK

Waterfowl zoochory is likely to be an important dispersal mechanism for many of the native plants we recorded (Green et al., 2016; Green & Elmberg, 2014). Waterfowl endozoochory facilitates long‐distance dispersal (LDD) between wetland catchments, whereas many plants we recorded are assigned to abiotic dispersal syndromes that imply no ability to disperse between catchments (see also Green et al., 2022; Urgyán et al., 2023). For example, given the high germination rates recorded, waterfowl likely contribute to the rapid expansion of *Carex pendula* within Britain in recent decades, including a pronounced northerly expansion (Preston et al., 2002). We recorded many species as a single seed, and increasing sampling effort would likely detect many additional plant species whose seeds are carried in waterfowl guts. These vectors may be vital to allow plants to modify their distributions in response to global change (Lovas‐Kiss et al., 2023; Nuñez et al., 2023; Urgyán et al., 2023).

### 4.2 Zoochory of alien species

All 56 non‐native propagules were recovered from feces from urban environments. In urban areas, many non‐native plants are introduced deliberately or spread from gardens, and birds feeding in these areas are likely to disperse non‐native seeds to natural areas, promoting biological invasions. Spread of alien plants can also interfere with dispersal interactions between avian vectors and native plants (Costa et al., 2022; van Leeuwen, 2018). Although Canada geese can promote the dispersal and expansion of non‐native grasses in North America (Best & Arcese, 2009), we found no evidence that this alien goose was more likely to disperse alien plants in the UK than the native duck.

Among the dispersed plants (including native species) we recorded, there are many that are planted in gardens and parks. Some of these species spread easily when they are released into nature, impacting the native flora. For example, *Carex pendula* is native to the UK, but invasive in riparian areas in the USA (United States Department of Agriculture, 2013).

Among the four aliens whose dispersal we detected, *Rhus typhina* is native to North America, was cultivated in Britain by 1629, and is popular in gardens (Preston et al., 2002). *Lepidium dydimus*, of South American origin, is a widespread alien in both hemispheres (Preston et al., 2002). *Kickxia spuria* is an archaeophyte in Britain, native to Europe and Asia, is introduced to other continents, and sometimes considered a noxious weed (Preston et al., 2002).

*Lemna minuta*, native to the Americas, was first recorded in Britain in 1977, and has spread rapidly since the late 1980s, partly through human vectors (Preston et al., 2002; Stace & Crawley, 2015). There is evidence that it is displacing native *Lemna* species, and can also colonize sites unsuitable for them, with impacts on aquatic biodiversity (Ceschin et al., 2020). It is likely to be readily dispersed by waterfowl epizoochory (Coughlan et al., 2015), although our results suggest that endozoochory may also be an important dispersal mechanism, as supported by the recent recovery of viable native *Lemna* from feces of mute swans (Paolacci et al., 2023). Closely related *Wolffia* can also be dispersed by waterfowl endozoochory (Silva et al., 2018).

Ducks and geese will likely be dispersing many more alien plant species in the UK than those identified in our study, together with alien aquatic invertebrates (Green, 2016; Green et al., 2023).

### 4.3 Differences between dabbling ducks and geese in dispersed plants and their traits

The dispersed plant community was significantly different between mallards and Canada geese (Figure 2). We found differences between bird species in the dispersal syndromes of seeds. Abiotic dispersal mechanisms have been shown to provide lower median and maximum dispersal distances than endozoochory (Bullock et al., 2017). Widespread assumptions that morphological dispersal syndromes directly reflect dispersal patterns in nature appear to be fundamentally flawed, and have been reinforced by the lack of field studies of zoochory by non‐frugivores (Green et al., 2022). Nevertheless, dispersal syndromes can be reinterpreted as traits giving an insight into how diaspores are ingested by waterfowl. The frequencies of barochory/autochory and hydrochory were higher in mallards, whereas epizoochory and anemochory were dominant in geese. The high endozoochory rates we observed for seeds assigned to epizoochory syndromes is similar to previous findings for dabbling ducks (Green et al., 2022) and geese (Navarro‐Ramos et al., 2024). How and why waterfowl ingest seeds with hooks or other traits used to assign the epizoochory syndrome deserves further investigation.

Differences between the two birds in feeding microhabitats along the aquatic‐terrestrial gradient may largely explain these differences in syndromes, since e.g., the hydrochory syndrome is associated with aquatic plants, and anemochory with terrestrial plants. Ellenberg *F* values of dispersed plant species show that mallards generally dispersed seeds with higher moisture requirements, while Canada geese did not disperse submerged plants. This is consistent with the more aquatic feeding habitats of dabbling ducks and the more regular terrestrial grazing of geese (see also Almeida et al., 2022).

We also found that mallards disperse larger seeds on average than Canada geese. The largely granivorous mallards may target larger, nutritious seeds, whereas herbivorous geese are more likely to ingest smaller seeds inadvertently with green parts of terrestrial plants. However, geese can also intentionally filter floating seeds from the water surface (personal observation). Larger seeds tend to have longer gut retention times, making LDD by endozoochory more likely (García‐Álvarez et al., 2015; Lovas‐Kiss et al., 2020). Bill morphology is also relevant to seed size, and mallards generally disperse larger seeds on average than those dispersed by teal *Anas crecca* (a smaller dabbling duck) feeding in the same habitats, since teal have a higher density of bill lamellae used for filtering (Green et al., 2016).

Each sample we analyzed contained only a small fraction of daily faecal production (Hahn et al., 2008), so our results imply high rates of endozoochory by both bird species. Given its greater abundance, total biomass and mobility, mallards are likely to be the more important vector in the UK overall (Brides et al., 2023; Frost et al., 2019). Nevertheless, the biomass of Canada geese can exceed that of mallards in urban habitats, and they are likely to be more important for dispersal of many terrestrial plants between urban sites, and from urban to natural habitats.

As in most of northern Europe, the greylag goose *Anser anser* is also widespread in the UK, with a mixture of migratory and feral birds (Frost et al., 2019). Greylag and Canada geese often form mixed flocks and feed in a similar manner. We may expect these two geese species to disperse similar sets of plants, although this is a topic for future research. Other dabbling duck species become abundant in our study area during the wintering period. Coexisting dabbling ducks disperse similar sets of plants (Sebastián‐González et al., 2020), and our results indicate that a combination of one dabbling duck and one goose are likely to disperse a larger set of plants than those dispersed by two duck or two geese species (see also Almeida et al., 2022). The patterns we detected may thus be relevant across much of the northern hemisphere where mallards and geese (often greylags or Canada geese) are the dominant resident waterfowl.

### 4.4 Differences in the dispersed plants and their traits between urban and natural sites

Rarefaction analysis shows that while mallards disperse more plant species than Canada geese in natural environments, there is no such difference in urban environments (Figure S3). Differences in feeding habits between species may be reduced in the more homogeneous urban habitats. We also found differences between habitats in the dispersal syndromes of plants dispersed. The proportion of plant species with an epizoochory syndrome was higher in natural habitats, whereas the frequencies of hydrochory and anemochory were higher in urban environments. Moreover, the fruit types of dispersed seeds differed, with capsules dominating in natural habitats, and achenes in urban environments (Figure 3). More seeds with higher Ellenberg *F* values (i.e., shoreline and fully aquatic plant species) were dispersed in urban environments (Figure 3).

These findings for syndromes, moisture requirements, and fruit types are interrelated, e.g., fully aquatic plants are more likely to have hydrochory syndromes and achenes. This overall pattern may partly be due to high rates of disturbance in urban environments, notably from dog‐walking, since people and their dogs regularly scare birds onto water. Urban sites also typically offer a more limited area of suitable grazing habitat bordered by roads or housing, and these terrestrial habitats are often frequently mown to provide lawns attractive to the public (personal observations). This mowing may reduce seed availability to grazing birds. Furthermore, urban wetlands are artificially constructed and often have shallow, relatively stable bathymetry providing good habitat for submerged plants. Finally, they are typically eutrophic, favouring plants such as *P. pectinatus* and *L. minor*.

### 4.5 Seasonality

Endozoochory rates were generally higher in the second half of the year, as expected given the phenology of production of diaspores from angiosperms in England. Since the peak in endozoochory rates in late summer was not strongly pronounced, instead continuing into autumn, our results also suggest that endozoochory can occur at high rates for months after seed production, as shown for ducks in central and southern Europe (Brochet et al., 2010; Figuerola et al., 2003; Urgyán et al., 2023). Hence, seed dispersal by waterfowl is not strongly coupled to the phenology of fruit production in a manner comparable to frugivory (González‐Varo et al., 2021), and this likely favours poleward plant dispersal in response to climate change (Lovas‐Kiss et al., 2023; Urgyán et al., 2023).

## 5 CONCLUSIONS

Our study demonstrates the importance of seed dispersal by widespread species of ducks and geese for plant species with different traits. Most of these species were previously assumed to lack mechanisms for LDD or dispersal between isolated habitat patches. The trait composition of species dispersed by waterfowl endozoochory varies both between vectors and between urban and natural habitats. Seed size and plant moisture requirements are diagnostic traits, as are fruit type and those aspects of diaspore morphology used to define popular dispersal syndromes. Future research should compare the traits of seeds dispersed with those available in the environment and aim to clarify how “other syndromes” can predict spatial and interspecific variation in endozoochory. More broadly, a variety of approaches (e.g., movement ecology, network studies, plant establishment experiments) are needed to advance our understanding of the influence of waterfowl endozoochory on the structure and species composition of local vegetation, and on plant distributions at a broader scale (see Green et al., 2023 for review).

## AUTHOR CONTRIBUTIONS

**Pál Tóth:** Conceptualization (equal); formal analysis (equal); visualization (equal); writing – original draft (equal); writing – review and editing (equal). **Andy J. Green:** Conceptualization (equal); data curation (equal); writing – original draft (equal); writing – review and editing (equal). **David M. Wilkinson:** Data curation (equal); methodology (equal); writing – review and editing (equal). **Kane Brides:** Data curation (equal); methodology (equal); writing – review and editing (equal). **Ádám Lovas‐Kiss:** Conceptualization (equal); data curation (equal); formal analysis (equal); methodology (equal); writing – original draft (equal); writing – review and editing (equal).

## CONFLICT OF INTEREST STATEMENT

Authors have no conflict of interest to declare.

## Supporting information


Appendix S1.

## Data Availability

All the data are available at: https://doi.org/10.6084/m9.figshare.24242524.v1.

## References

[ece310677-bib-0001] Almeida, B. A. , Lukács, B. A. , Lovas‐Kiss, Á. , Reynolds, C. , & Green, A. J. (2022). Functional traits drive dispersal interactions between European waterfowl and seeds. Frontiers in Plant Science, 12, 795288. 10.3389/fpls.2021.795288 35173751 PMC8843038

[ece310677-bib-0002] Almeida, B. A. , Sebastián‐González, E. , dos Anjos, L. , & Green, A. J. (2020). Comparing the diversity and composition of waterbird functional traits between natural, restored and artificial wetlands. Freshwater Biology, 65, 2196–2210. 10.1111/fwb.13618

[ece310677-bib-0003] Bates, D. , Maechler, M. , Bolker, B. M. , & Walker, S. (2015). Fitting linear mixed‐effects models using lme4. Journal of Statistical Software, 67, 1–48. 10.18637/jss.v067.i01

[ece310677-bib-0004] Best, R. J. , & Arcese, P. (2009). Exotic herbivores directly facilitate the exotic grasses they graze: Mechanisms for an unexpected positive feedback between invaders. Oecologia, 159, 139–150. 10.1007/s00442-008-1172-1 18941792

[ece310677-bib-0005] Bird Life International . (2023). Species factsheet: *Anas platyrhynchos* . http://datazone.birdlife.org/species/factsheet/mallard‐anas‐platyrhynchos

[ece310677-bib-0006] Bojnanský, V. , & Fargašová, A. (2007). Atlas of seeds and fruits of central and east‐European flora: The Carpathian Mountains region. Springer.

[ece310677-bib-0007] Brides, K. , Wood, K. A. , Barbour, J. , Petrek, S. W. , Cooper, J. , Leighton, K. , & Grogan, A. (2023). Moult migration, site fidelity and survival of Canada Geese Branta canadensis at Windermere, Cumbria. Wildflowl, 73, 43–63.

[ece310677-bib-0008] Brochet, A. L. , Guillemain, M. , Fritz, H. , Gauthier‐Clerc, M. , & Green, A. J. (2010). Plant dispersal by teal (*Anas crecca*) in the Camargue: Duck guts are more important than their feet. Freshwater Biology, 55, 1262–1273. 10.1111/j.1365-2427.2009.02350.x

[ece310677-bib-0009] Brooks, M. E. , Kristensen, K. , Van Benthem, K. J. , Magnusson, A. , Berg, C. W. , Nielsen, A. , Skaug, H. J. , Mächler, M. , & Bolker, B. M. (2017). glmmTMB balances speed and flexibility among packages for zero‐inflated generalized linear mixed modeling. The R Journal, 9(2), 378–400. 10.3929/ethz-b-000240890

[ece310677-bib-0010] Bullock, J. M. , Mallada González, L. , Tamme, R. , Götzenberger, L. , White, S. M. , Pärtel, M. , & Hooftman, D. A. (2017). A synthesis of empirical plant dispersal kernels. Journal of Ecology, 105(1), 6–19. 10.1111/1365-2745.12666

[ece310677-bib-0011] Campbell, D. A. , Cole, C. A. , & Brooks, R. P. (2002). A comparison of created and natural wetlands in Pennsylvania, USA. Wetlands Ecology and Management, 10, 41–49. 10.1023/A:1014335618914

[ece310677-bib-0012] Cappers, R. T. J. , Bekker, R. M. , & Jans, J. E. A. (2012). Digitale Zadenatlas van Nederland/Digital seed atlas of the Netherlands (Vol. 4). Barkhuis.

[ece310677-bib-0013] Ceschin, S. , Ferrante, G. , Mariani, F. , Traversetti, L. , & Ellwood, N. T. W. (2020). Habitat change and alteration of plant and invertebrate communities in waterbodies dominated by the invasive alien macrophyte *Lemna minuta* Kunth. Biological Invasions, 22, 1325–1337. 10.1007/s10530-019-02185-5

[ece310677-bib-0014] Chao, A. , Gotelli, N. J. , Hsieh, T. C. , Sander, E. L. , Ma, K. H. , Colwell, R. K. , & Ellison, A. M. (2014). Rarefaction and extrapolation with hill numbers: A framework for sampling and estimation in species diversity studies. Ecological Monographs, 84(1), 45–67. 10.1890/13-0133.1

[ece310677-bib-0015] Costa, A. , Heleno, R. , Dufrene, Y. , Huckle, E. , Gabriel, R. , Harrison, X. , Schabo, D. G. , Farwig, N. , & Kaiser‐Bunbury, C. N. (2022). Seasonal variation in impact of non‐native species on tropical seed dispersal networks. Functional Ecology, 36, 2713–2726. 10.1111/1365-2435.14171

[ece310677-bib-0016] Coughlan, N. E. , Kelly, T. C. , & Jansen, M. A. K. (2015). Mallard duck (*Anas platyrhynchos*)‐mediated dispersal of Lemnaceae: A contributing factor in the spread of invasive *Lemna minuta*? Plant Biology, 17, 108–114. 10.1111/plb.12182 24802728

[ece310677-bib-0017] Cruz, J. C. , Ramos, J. A. , da Silva, L. P. , Tenreiro, P. Q. , & Heleno, R. H. (2013). Seed dispersal networks in an urban novel ecosystem. European Journal of Forest Research, 132, 887–897. 10.1007/s10342-013-0722-1

[ece310677-bib-0018] Daru, B. H. , Davies, T. J. , Willis, C. G. , Meineke, E. K. , Ronk, A. , Zobel, M. , Pärtel, M. , Antonelli, A. , & Davis, C. C. (2021). Widespread homogenization of plant communities in the Anthropocene. Nature Communications, 12, 6983. 10.1038/s41467-021-27186-8 PMC8648934 34873159

[ece310677-bib-0019] Dessborn, L. , Brochet, A. L. , Elmberg, J. , Legagneux, P. , Gauthier‐Clerc, M. , & Guillemain, M. (2011). Geographical and temporal patterns in the diet of pintail *Anas acuta*, wigeon *Anas penelope*, mallard *Anas platyrhynchos* and teal *Anas crecca* in the Western Palearctic. European Journal of Wildlife Research, 57, 1119–1129. 10.1007/s10344-011-0577-z

[ece310677-bib-0020] Domina, G. , Galasso, G. , Bartolucci, F. , & Guarino, R. (2018). Ellenberg indicator values for the vascular flora alien to Italy. Flora Mediterranea, 28, 53–61. 10.7320/FlMedit28.053.1

[ece310677-bib-0022] Emer, C. , Galetti, M. , Pizo, M. A. , Jordano, P. , & Verdú, M. (2019). Defaunation precipitates the extinction of evolutionarily distinct interactions in the Anthropocene. Science Advances, 5(6), eaav6699. 10.1126/sciadv.aav6699 31223648 PMC6584213

[ece310677-bib-0023] Evans, T. , Blackburn, T. M. , Jeschke, J. M. , Probert, A. F. , & Bacher, S. (2020). Application of the socio‐economic impact classification for alien taxa (SEICAT) to a global assessment of alien bird impacts. NeoBiota, 62, 123–142. 10.3897/neobiota.62.51150

[ece310677-bib-0024] Evans, T. , Jeschke, J. M. , Liu, C. , Redding, D. W. , Şekercioğlu, Ç. H. , & Blackburn, T. M. (2021). What factors increase the vulnerability of native birds to the impacts of alien birds? Ecography, 44(5), 727–739. 10.1111/ecog.05000

[ece310677-bib-0025] Figuerola, J. , Green, A. J. , & Santamaría, L. (2003). Passive internal transport of aquatic organisms by waterfowl in Doñana, south‐West Spain. Global Ecology and Biogeography, 12(5), 427–436. 10.1046/j.1466-822X.2003.00043.x

[ece310677-bib-0026] Forget, P.‐M. , Jordano, P. , Lambert, J. E. , Böhning‐Gaese, K. , Traveset, A. , & Wright, S. J. (2011). Frugivores and seed dispersal (1985–2010); the ‘seeds’ dispersed, established and matured. Acta Oecologica, 37, 517–520. 10.1016/j.actao.2011.09.008

[ece310677-bib-0027] Frost, T. , Austin, G. , Hearn, R. , McAvoy, S. , Robinson, A. , Stroud, D. , Woodward, I. , & Wotton, S. (2019). Population estimates of wintering waterbirds in Great Britain. British Birds, 112, 130–145.

[ece310677-bib-0028] Funk, J. L. , Larson, J. E. , Ames, G. M. , Butterfield, B. J. , Cavender‐Bares, J. , Firn, J. , Laughlin, D. C. , Sutton‐Grier, A. E. , Williams, L. , & Wright, J. (2017). Revisiting the Holy G rail: Using plant functional traits to understand ecological processes. Biological Reviews, 92(2), 1156–1173. 10.1111/brv.12275 27103505

[ece310677-bib-0029] García‐Álvarez, A. , van Leeuwen, C. H. , Luque, C. J. , Hussner, A. , Vélez‐Martín, A. , Pérez‐Vázquez, A. , Green, A. J. , & Castellanos, E. M. (2015). Internal transport of alien and native plants by geese and ducks: An experimental study. Freshwater Biology, 60(7), 1316–1329. 10.1111/fwb.12567

[ece310677-bib-0030] González‐Varo, J. P. , Rumeu, B. , Albrecht, J. , Arroyo, J. M. , Bueno, R. S. , Burgos, T. , da Silva, L. P. , Escribano‐Ávila, G. , Farwig, N. , García, D. , Heleno, R. H. , Illera, J. C. , Jordano, P. , Kurek, P. , Simmons, B. I. , Virgós, E. , Sutherland, W. J. , & Traveset, A. (2021). Limited potential for bird migration to disperse plants to cooler latitudes. Nature, 595(7865), 75–79. 10.1038/s41586-021-03665-2 34163068

[ece310677-bib-0031] Green, A. J. (2016). The importance of waterbirds as an overlooked pathway of invasion for alien species. Diversity and Distributions, 22, 239–247. 10.1111/ddi.12392

[ece310677-bib-0032] Green, A. J. , Baltzinger, C. , & Lovas‐Kiss, Á. (2022). Plant dispersal syndromes are unreliable, especially for predicting zoochory and long‐distance dispersal. Oikos, e08327. 10.1111/oik.08327

[ece310677-bib-0033] Green, A. J. , & Elmberg, J. (2014). Ecosystem services provided by waterbirds. Biological Reviews, 89(1), 105–122. 10.1111/brv.12045 23786594

[ece310677-bib-0034] Green, A. J. , Lovas‐Kiss, Á. , Reynolds, C. , Sebastián‐González, E. , Silva, G. G. , van Leeuwen, C. H. A. , & Wilkinson, D. M. (2023). Dispersal of aquatic and terrestrial organisms by waterbirds: A review of current knowledge and future priorities. Freshwater Biology, 68, 173–190. 10.1111/fwb.14038

[ece310677-bib-0035] Green, A. J. , Lovas‐Kiss, Á. , Stroud, R. A. , Tierney, N. , & Fox, A. D. (2018). Plant dispersal by Canada geese in Arctic Greenland. Polar Research, 37(1), 1508268. 10.1080/17518369.2018.1508268

[ece310677-bib-0036] Green, A. J. , Soons, M. B. , Brochet, A. L. , & Kleyheeg, E. (2016). In Ç. H. Şekercioğlu , D. G. Wenny , & C. J. Whelan (Eds.), Dispersal of plants by waterbirds. Why birds matter: Avian ecological function and ecosystem services (pp. 147–195). The University of Chicago Press. 10.7208/Chicago/9780226382777.001.0001

[ece310677-bib-0037] Hagemeijer, E. J. M. , & Blair, M. J. (Eds.). (1997). The EBCC Atlas of European breeding birds: Their distribution and abundance. T & A D Poyser.

[ece310677-bib-0038] Hahn, S. , Bauer, S. , & Klaassen, M. (2008). Quantification of allochthonous nutrient input into freshwater bodies by herbivorous waterbirds. Freshwater Biology, 53, 181–193. 10.1111/j.1365-2427.2007.01881.x

[ece310677-bib-0039] Hattermann, D. , Bernhardt‐Römermann, M. , Otte, A. , & Eckstein, R. L. (2019). Geese are overlooked dispersal vectors for vascular plants in archipelago environments. Journal of Vegetation Science, 30(3), 533–541. 10.1111/jvs.12742

[ece310677-bib-0021] Hill, M. O. , Mountford, J. O. , Roy, D. B. , & Bunce, R. G. H. (1999). Ellenberg's indicator values for British plants. ECOFACT vol. 2 Technical Annex, Inst. of Terrestrial Ecology.

[ece310677-bib-0040] Hui, F. K. (2016). Boral–Bayesian ordination and regression analysis of multivariate abundance data in R. Methods in Ecology and Evolution, 7(6), 744–750. 10.1111/2041-210X.12514

[ece310677-bib-0041] Hui, F. K. C. (2018). boral: Bayesian Ordination and Regression AnaLysis. R package version 1.7.

[ece310677-bib-0042] Jansson, K. , Josefsson, M. , & Weidema, I. (2008). NOBANIS – Invasive Alien Species Fact Sheet–Branta canadensis. From: Online Database of the North European and Baltic Network on Invasive Alien Species – NOBANIS. www.nobanis.org

[ece310677-bib-0043] Julve, P. (1998). Baseflor. Index botanique, écologique et chorologique de la flore de France. Inst. Catholique de Lille, Lille, France. http://perso.wanadoo.fr/philippe.julve/catminat.htm

[ece310677-bib-0044] Kawakami, K. , Mizusawa, L. , & Higuchi, H. (2009). Re‐established mutualism in a seed‐dispersal system consisting of native and introduced birds and plants on the Bonin Islands, Japan. Ecological Research, 24, 741–748. 10.1007/s11284-008-0543-8

[ece310677-bib-0045] Kear, J. (Ed.). (2005). Ducks, geese and swans: Species accounts (Cairina to Mergus) (Vol. 2). Oxford University Press.

[ece310677-bib-0046] Kleyheeg, E. , Treep, J. , De Jager, M. , Nolet, B. A. , & Soons, M. B. (2017). Seed dispersal distributions resulting from landscape‐dependent daily movement behaviour of a key vector species, *Anas platyrhynchos* . Journal of Ecology, 105, 1279–1289. 10.1111/1365-2745.12738

[ece310677-bib-0047] La Rosa, A. M. , Smith, C. W. , & Gardner, D. E. (1985). Role of alien and native birds in the dissemination of Firetree (*Myrica faya* Ait.‐Myricaceae) and associated plants in Hawaii. Pacific Science, 39(4), 372–378.

[ece310677-bib-0048] Lovas‐Kiss, Á. , Martín‐Vélez, V. , Brides, K. , Wilkinson, D. M. , Griffin, L. R. , & Green, A. J. (2023). Migratory geese allow plants to disperse to cooler latitudes across the ocean. Journal of Biogeography, 50, 1602–1614. 10.1111/jbi.14674

[ece310677-bib-0049] Lovas‐Kiss, Á. , Vincze, O. , Löki, V. , Pallér‐Kapusi, F. , Halasi‐Kovács, B. , Kovács, G. , Green, A. J. , & Lukács, B. A. (2020). Experimental evidence of dispersal of invasive cyprinid eggs inside migratory waterfowl. Proceedings of the National Academy of Sciences, 117(27), 15397–15399. 10.1073/pnas.2004805117 PMC7355035 32571940

[ece310677-bib-0050] Lovas‐Kiss, Á. , Vizi, B. , Vincze, O. , Molnár, V. A. , & Green, A. J. (2018). Endozoochory of aquatic ferns and angiosperms by mallards in Central Europe. Journal of Ecology, 106(4), 1714–1723. 10.1111/1365-2745.12913

[ece310677-bib-0051] Ma, Z. , Cai, Y. , Li, B. , & Chen, J. (2010). Managing wetland habitats for waterbirds: An international perspective. Wetlands, 30, 15–27. 10.1007/s13157-009-0001-6

[ece310677-bib-0052] Martin‐Albarracin, V. L. , Nuñez, M. A. , & Amico, G. C. (2018). Non‐redundancy in seed dispersal and germination by native and introduced frugivorous birds: Implications of invasive bird impact on native plant communities. Biodiversity and Conservation, 27, 3793–3806. 10.1007/s10531-018-1629-4

[ece310677-bib-0053] Mooney, H. A. , & Cleland, E. E. (2001). The evolutionary impact of invasive species. Proceedings of the National Academy of Sciences USA, 98, 5446–5451. 10.1073/pnas.091093398 PMC33232 11344292

[ece310677-bib-0054] Murray, C. G. , Kasel, S. , Loyn, R. H. , Hepworth, G. , & Hamilton, A. J. (2013). Waterbird use of artificial wetlands in an Australian urban landscape. Hydrobiologia, 716, 131–146. 10.1007/s10750-013-1558-x

[ece310677-bib-0077] Navarro‐Ramos, M. J. , van Leeuwen, C. H. A. , Olsson, C. , Elmberg, J. , Månsson, J. , Martín‐Vélez, V. , Lovas‐Kiss, A. , & Green, A. J. (2024). Seed dispersal between aquatic and agricultural habitats by greylag geese. Agriculture, Ecosystems & Environment, 359, 108741. 10.1016/j.agee.2023.108741

[ece310677-bib-0055] Navedo, J. G. , Masero, J. A. , Sanchez‐Guzman, J. M. , Abad‐Gomez, J. M. , Gutierrez, J. S. , Sanson, E. G. , Villegas, A. , Costillo, E. , Corbacho, C. , & Moran, R. (2012). International importance of Extremadura, Spain, for overwintering migratory dabbling ducks: A role for reservoirs. Bird Conservation International, 22, 316–327. 10.1017/S0959270911000311

[ece310677-bib-0056] Nuñez, T. A. , Prugh, L. R. , & Hille Ris Lambers, J. (2023). Animal‐mediated plant niche tracking in a changing climate. Trends in Ecology & Evolution, 38, 654–665. 10.1016/j.tree.2023.02.005 36932024

[ece310677-bib-0057] Paolacci, S. , Jansen, M. A. K. , Stejskal, V. , Kelly, T. C. , & Coughlan, N. E. (2023). Metabolically active angiosperms survive passage through the digestive tract of a large‐bodied waterbird. Royal Society Open Science, 10, 230090. 10.1098/rsos.230090 36968238 PMC10031429

[ece310677-bib-0058] Pesendorfer, M. B. , Sillett, T. S. , Koenig, W. D. , & Morrison, S. A. (2016). Scatter‐hoarding corvids as seed dispersers for oaks and pines: A review of a widely distributed mutualism and its utility to habitat restoration. The Condor, 118(2), 215–237. 10.1650/CONDOR-15-125.1

[ece310677-bib-0059] Preston, C. D. , Pearman, D. A. , & Dines, T. D. (2002). New Atlas of the British and Irish Flora. Oxford University Press.

[ece310677-bib-0060] R Core Team . (2021). R: A language and environment for statistical computing. R Foundation for Statistical Computing, Vienna, Austria. https://www.R‐project.org/

[ece310677-bib-0061] Reyns, N. , Casaer, J. , De Smet, L. , Devos, K. , Huysentruyt, F. , Robertson, P. A. , Verbeke, T. , & Adriaens, T. (2018). Cost‐benefit analysis for invasive species control: The case of greater Canada goose *Branta canadensis* in Flanders (Northern Belgium). PeerJ, 6, e4283. 10.7717/peerj.4283 29404211 PMC5793711

[ece310677-bib-0062] Robinson, R. A. , Leech, D. I. , & Clark, J. A. (2021). The Online Demography Report: Bird ringing and nest recording in Britain & Ireland in 2020. BTO, Thetford (http://www.bto.org/ringing‐report, created on 7‐August‐2021).

[ece310677-bib-0063] Schneiberg, I. , Boscolo, D. , Devoto, M. , Marcilio‐Silva, V. , Dalmaso, C. A. , Ribeiro, J. W. , Ribeiro, M. C. , Guaraldo, A. D. C. , Niebuhr, B. B. , & Varassin, I. G. (2020). Urbanization homogenizes the interactions of plant‐frugivore bird networks. Urban Ecosystems, 23(3), 457–470. 10.1007/s11252-020-00927-1

[ece310677-bib-0064] Sebastián‐González, E. , Lovas‐Kiss, Á. , Soons, M. B. , van den Broek, B. , & Green, A. J. (2020). Waterbird seed‐dispersal networks are similarly nested but less modular than those of frugivorous birds, and not driven by functional traits. Functional Ecology, 34(11), 2283–2291. 10.1111/1365-2435.13657

[ece310677-bib-0065] Silva, G. G. , Green, A. J. , Weber, V. , Hoffmann, P. , Lovas‐Kiss, Á. , Stenert, C. , & Maltchik, L. (2018). Whole angiosperms *Wolffia columbiana* disperse by gut passage through wildfowl in South America. Biology Letters, 14(12), 20180703. 10.1098/rsbl.2018.0703 30958251 PMC6303509

[ece310677-bib-0066] Soons, M. B. , Brochet, A. L. , Kleyheeg, E. , & Green, A. J. (2016). Seed dispersal by dabbling ducks: An overlooked dispersal pathway for a broad spectrum of plant species. Journal of Ecology, 104(2), 443–455. 10.1111/1365-2745.12531

[ece310677-bib-0067] Stace, C. A. , & Crawley, M. J. (2015). Alien plants. Collins.

[ece310677-bib-0068] Traveset, A. , Heleno, R. , & Nogales, M. (2014). The ecology of seed dispersal. In R. S. Gallaguer (Ed.), Seeds. The ecology of regeneration in plant communities (Vol. 3, pp. 62–93). CAB International. 10.1079/978117880641836.0062

[ece310677-bib-0069] United States Department of Agriculture . (2013). Weed Risk Assessment for *Carex pendula* Huds. (Cyperaceae) – Pendulous sedge; Animal and Plant Health Inspection Service.

[ece310677-bib-0070] Urgyán, R. , Lukács, B. A. , Fekete, R. , Molnár, V. A. , Nagy, A. , Orsolya, V. , Green, A. J. , & Lovas‐Kiss, Á. (2023). Plants dispersed by a non‐frugivorous migrant change throughout the annual cycle. Global Ecology and Biogeography, 32, 70–82. 10.1111/geb.13608

[ece310677-bib-0071] Van der Pijl, L. (1982). Principles of dispersal in higher plants (Vol. 214). Springer‐Verlag.

[ece310677-bib-0072] van Leeuwen, C. H. A. (2018). Internal and external dispersal of plants by animals: An aquatic perspective on alien interference. Frontiers in Plant Science, 9, 153. 10.3389/fpls.2018.00153 29487609 PMC5816930

[ece310677-bib-0073] Vizentin‐Bugoni, J. , Tarwater, C. E. , Foster, J. T. , Drake, D. R. , Gleditsch, J. M. , Hruska, A. M. , Kelley, J. P. , & Sperry, J. H. (2019). Structure, spatial dynamics, and stability of novel seed dispersal mutualistic networks in Hawai'i. Science, 364(6435), 78–82. 10.1126/science.aau8751 30948550

[ece310677-bib-0074] Wang, Y. I. , Naumann, U. , Wright, S. T. , & Warton, D. I. (2012). Mvabund–an R package for model‐based analysis of multivariate abundance data. Methods in Ecology and Evolution, 3(3), 471–474. 10.1111/j.2041-210X.2012.00190.x

[ece310677-bib-0075] Wernham, C. , Toms, M. , Marchant, J. , Clark, J. , Siriwardena, G. , & Baillie, S. (2002). The migration breeding atlas: Movements of the birds of Britain and Ireland. T & AD Poyser.

[ece310677-bib-0076] Yalden, D. W. , & Albarella, U. (2009). The history of British birds. Oxford University Press.

