# Supplementary material for: Plant traits associated with seed dispersal by ducks and geese in urban and natural habitats
Source: Ecol Evol. 2023 Nov 22;13(11):e10677. doi: 10.1002/ece3.10677 (PMC10663722; doi:10.1002/ece3.10677)

**Table S1.** Details of faecal samples collected from two different waterbird species from eighteen locations (see also Fig. 1 and Tab. 1)

| **date** | **location** | **WGS84_X** | **WGS84_Y** | **type of wetlands** | **species** | **faecal samples** |
| --- | --- | --- | --- | --- | --- | --- |
| 25 January 2016 | Martin Mere | 53.622457 | -2.868174 | natural | *Anas platyrhynchos* | 2 |
| 8 May 2016 | Moses Gate Country Park | 53.556275 | -2.391455 | urban | *Anas platyrhynchos* | 1 |
| 8 May 2016 | Moses Gate Country Park | 53.556275 | -2.391455 | urban | *Branta canadensis* | 17 |
| 16 May 2016 | Rostherne Mere | 53.353957 | -2.385902 | natural | *Anas platyrhynchos* | 19 |
| 16 May 2016 | Rostherne Mere | 53.353957 | -2.385902 | natural | *Branta canadensis* | 2 |
| 21 June 2016 | Sefton Park Liverpool | 53.382314 | -2.938112 | urban | *Anas platyrhynchos* | 5 |
| 22 June 2016 | Sefton Park Liverpool | 53.382314 | -2.938112 | urban | *Anas platyrhynchos* | 4 |
| 22 June 2016 | Sefton Park Liverpool | 53.382314 | -2.938112 | urban | *Branta canadensis* | 13 |
| 23 June 2016 | Moses Gate Country Park | 53.556275 | -2.391455 | urban | *Anas platyrhynchos* | 5 |
| 23 June 2016 | Moses Gate Country Park | 53.556275 | -2.391455 | urban | *Branta canadensis* | 10 |
| 23 June 2016 | Sefton Park Liverpool | 53.382314 | -2.938112 | urban | *Branta canadensis* | 3 |
| 26 June 2016 | Fell Foot | 54.27475 | -2.95362 | natural | *Anas platyrhynchos* | 22 |
| 26 June 2016 | Ambleside | 54.421109 | -2.964799 | natural | *Branta canadensis* | 17 |
| 26 June 2016 | Bowness on Windermere | 54.365353 | -2.924245 | natural | *Branta canadensis* | 11 |
| 26 June 2016 | Fell Foot | 54.27475 | -2.95362 | natural | *Branta canadensis* | 11 |
| 29 June 2016 | Fell Foot | 54.27475 | -2.95362 | natural | *Anas platyrhynchos* | 7 |
| 5 July 2016 | Sefton Park Liverpool | 53.382314 | -2.938112 | urban | *Anas platyrhynchos* | 24 |
| 5 July 2016 | Stanley Park Liverpool | 53.436656 | -2.965542 | urban | *Anas platyrhynchos* | 6 |
| 5 July 2016 | Liverpool-Leeds Canal (Liverpool) | 53.424039 | -2.989574 | urban | *Branta canadensis* | 6 |
| 14 July 2016 | Budworth Mere | 53.287386 | -2.517185 | natural | *Anas platyrhynchos* | 5 |
| 14 July 2016 | Pickmere | 53.289836 | -2.476781 | natural | *Anas platyrhynchos* | 15 |
| 14 July 2016 | Rostherne Mere | 53.353957 | -2.385902 | natural | *Anas platyrhynchos* | 34 |
| 14 July 2016 | Budworth Mere | 53.287386 | -2.517185 | natural | *Branta canadensis* | 10 |
| 26 July 2016 | Sefton Park Liverpool | 53.382314 | -2.938112 | urban | *Anas platyrhynchos* | 15 |
| 26 July 2016 | Sefton Park Liverpool | 53.382314 | -2.938112 | urban | *Branta canadensis* | 5 |
| 29 July 2016 | Crosby Boating Lake | 53.474134 | -3.035841 | urban | *Branta canadensis* | 6 |
| 30 July 2016 | Crosby Boating Lake | 53.474134 | -3.035841 | urban | *Anas platyrhynchos* | 11 |
| 31 July 2016 | Stanley Park Liverpool | 53.436656 | -2.965542 | urban | *Anas platyrhynchos* | 18 |
| 2 August 2016 | West Kirby | 53.370353 | -3.179249 | natural | *Branta canadensis* | 6 |
| 3 August 2016 | Ellesmere Mere | 52.908355 | -2.883214 | natural | *Branta canadensis* | 9 |
| 16 August 2016 | Sefton Park Liverpool | 53.382314 | -2.938112 | urban | *Anas platyrhynchos* | 9 |
| 1 September 2016 | Sefton Park Liverpool | 53.382314 | -2.938112 | urban | *Anas platyrhynchos* | 15 |
| 23 September 2016 | Sefton Park Liverpool | 53.382314 | -2.938112 | urban | *Branta canadensis* | 5 |
| 13 September 2016 | Rostherne Mere | 53.353957 | -2.385902 | natural | *Anas platyrhynchos* | 25 |
| 10 October 2016 | Sefton Park Liverpool | 53.382314 | -2.938112 | urban | *Anas platyrhynchos* | 15 |
| 14 April 2019 | Doxey Marshes | 52.821177 | -2.146054 | natural | *Branta canadensis* | 30 |
| 14 April 2019 | Wolseley bridge | 52.785478 | -1.970121 | natural | *Branta canadensis* | 30 |
| 22 April 2019 | Bowness on Windermere | 54.365353 | -2.924245 | natural | *Branta canadensis* | 30 |
| 27 April 2019 | Killington | 54.314697 | -2.629783 | natural | *Branta canadensis* | 29 |

**Table S2.** A) Results from the binomial glmer (lme4 package), using the presence/absence of seeds per sample as the dependent variable, and bird species, site type and sample mass as independent variables. Collection date was included as a random factor. B) The results of the negative binomial glmTMB (glmmTMB package), using the total number of seeds per sample as the dependent variable, and bird species, site type and sample mass as independent variables. Collection date was included as a random factor. C) The results of the negative binomial glmTMB (glmmTMB package), using the number of species per sample as the dependent variable and species, site type and sample mass as independent variables. Collection date was included as a random factor.

| ***A)*** | ***Estimate*** | ***Std. Error*** | ***z-value*** | ***P-value*** |
| --- | --- | --- | --- | --- |
| (Intercept) | -3.365 | 0.755 | -4.457 | 8.32e-06 |
| species mallard | 0.885 | 0.464 | 1.908 | 0.056 |
| site type urban | 0.397 | 0.862 | 0.460 | 0.646 |
| sample mass | 0.123 | 0.046 | 2.675 | 0.008 |
| ***B)*** | ***Estimate*** | ***Std. Error*** | ***z-value*** | ***P-value*** |
| (Intercept) | -2.913 | 0.892 | -3.265 | 0.001 |
| species mallard | 1.850 | 0.502 | 3.689 | <0.001 |
| site type urban | 0.627 | 1.055 | 0.594 | 0.552 |
| sample mass | 0.146 | 0.056 | 2.598 | 0.009 |
| ***C)*** | ***Estimate*** | ***Std. Error*** | ***z-value*** | ***P-value*** |
| (Intercept) | -2.866 | 0.579 | -4.950 | 7.43e-07 |
| species mallard | 0.859 | 0.379 | 2.266 | 0.024 |
| site type urban | 0.223 | 0.646 | 0.345 | 0.730 |
| sample mass | 0.075 | 0.036 | 2.082 | 0.037 |

**Table S3. Description of the dispersal syndromes applied to plant species recorded in our study, according to van der Pijl (1982) and Julve (1998).**

| **Syndrome** | **Description** | **Putative adaptation** |
| --- | --- | --- |
| anemochory | dispersal by wind | dust, plumed, winged diaspores, balloons |
| autochory | dispersal by the plant itself | ballistic features: tension in dead tissues or living tissues to shoot out the seeds; or creeping diaspores: hygroscopic bristles of diaspores performing movements |
| barochory | dispersal by gravity only | heavy fruits or seeds with no other obvious adaptations |
| endozoochory | dispersal in the digestive tract of the animals | fruits with attractive, fleshy, edible parts, signalling colours when mature |
| epizoochory | dispersal on the outside of the animals | hooks and spines on the fruits |
| hydrochory | dispersal by rain or water currents | hydrophobic or floating seeds |

Julve, P. (1998): Baseflor. Index botanique, écologique et chorologique de la flore de France. – Inst. Catholique de Lille, Lille, France, <http://perso.wanadoo.fr/philippe.julve/catminat.htm> accessed 26 November 2017.

Van der Pijl, L. (1982): Principles of dispersal in higher plants (Vol. 214). Berlin: Springer-Verlag

**Table S4. Corine Land Cover area and percentages for the collection sites within a radius of 1 km. Natural sites are in italics.**

| **Location** | **Corine Land cover** | **Area (ha)** | **Percentage of the cover** |
| --- | --- | --- | --- |
| *Wolseley bridge* | Pastures | 232 | 76 |
|  | Discontinuous urban fabric | 13 | 4 |
|  | Sport and leisure facilities | 26 | 8 |
|  | Non-irrigated arable land | 9 | 3 |
|  | Broad-leaved forest | 4 | 1 |
|  | Coniferous forest | 5 | 2 |
|  | Mixed forest | 14 | 5 |
| *West Kirby* | Continuous urban fabric | 41 | 14 |
|  | Discontinuous urban fabric | 195 | 65 |
|  | Sport and leisure facilities | 3 | 1 |
|  | Intertidal flats | 63 | 21 |
| Stanley Park Liverpool | Discontinuous urban fabric | 118 | 39 |
|  | Continuous urban fabric | 103 | 34 |
|  | Green urban areas | 81 | 27 |
| Sefton Park Liverpool | Discontinuous urban fabric | 156 | 52 |
|  | Continuous urban fabric | 8 | 3 |
|  | Green urban areas | 138 | 46 |
| *Rostherne Mere* | Non-irrigated arable land | 82 | 27 |
|  | Road and rail networks and associated land | 12 | 4 |
|  | Pastures | 161 | 53 |
|  | Water bodies | 47 | 16 |
| *Pickmere* | Non-irrigated arable land | 175 | 58 |
|  | Pastures | 87 | 29 |
|  | Discontinuous urban fabric | 41 | 13 |
| Moses Gate Country Park | Discontinuous urban fabric | 126 | 42 |
|  | Sport and leisure facilities | 161 | 53 |
|  | Pastures | 15 | 5 |
| *Martin Mere* | Sport and leisure facilities | 158 | 52 |
|  | Non-irrigated arable land | 143 | 48 |
| Liverpool-Leeds Canal (Liverpool) | Discontinuous urban fabric | 156 | 52 |
|  | Port areas | 114 | 38 |
|  | Industrial or commercial units | 32 | 10 |
| *Killington* | Pastures | 164 | 55 |
|  | Mixed forest | 3 | 1 |
|  | Natural grasslands | 32 | 11 |
|  | Moors and heathland | 37 | 12 |
|  | Water bodies | 65 | 21 |
| *Fell Foot* | Water bodies | 25 | 8 |
|  | Pastures | 46 | 15 |
|  | Coniferous forest | 40 | 13 |
|  | Mixed forest | 187 | 62 |
|  | Transitional woodland-shrub | 2 | 1 |
| *Ellesmere Mere* | Discontinuous urban fabric | 47 | 16 |
|  | Sport and leisure facilities | 67 | 22 |
|  | Non-irrigated arable land | 36 | 12 |
|  | Pastures | 105 | 35 |
|  | Water bodies | 48 | 16 |
| *Doxey Marshes* | Pastures | 26 | 9 |
|  | Discontinuous urban fabric | 93 | 31 |
|  | Industrial or commercial units | 7 | 2 |
|  | Non-irrigated arable land | 177 | 59 |
| Crosby Boating Lake | Discontinuous urban fabric | 113 | 37 |
|  | Port areas | 95 | 31 |
|  | Green urban areas | 44 | 15 |
|  | Intertidal flats | 16 | 5 |
|  | Water bodies | 26 | 8 |
|  | Sea and ocean | 8 | 3 |
| *Budworth Mere* | Discontinuous urban fabric | 1 | 0 |
|  | Sport and leisure facilities | 32 | 10 |
|  | Non-irrigated arable land | 109 | 36 |
|  | Pastures | 70 | 23 |
|  | Broad-leaved forest | 53 | 17 |
|  | Water bodies | 38 | 13 |
| *Bowness on Windermere* | Discontinuous urban fabric | 98 | 33 |
|  | Sport and leisure facilities | 20 | 7 |
|  | Land principally occupied by agriculture, with significant areas of natural vegetation | 1 | 0 |
|  | Broad-leaved forest | 20 | 7 |
|  | Mixed forest | 23 | 8 |
|  | Water bodies | 138 | 46 |
| *Ambleside* | Water bodies | 71 | 24 |
|  | Discontinuous urban fabric | 51 | 17 |
|  | Pastures | 93 | 31 |
|  | Mixed forest | 72 | 24 |
|  | Moors and heathland | 13 | 4 |

To classify the naturalness of the sample collection sites, we examined the Corine Land cover areas within a 1 km radius circle around the central coordinate of the sampling places. The Corine Land Cover map was based on a 2018 database available online (<https://www.data.gov.uk/dataset/cd2c59e7-afd9-471d-a056-c5845619dcd7/corine-land-cover>-2018-for-the-uk-isle-of-man-jersey-and-guernsey?fbclid=IwAR2Gkec08sW8upDUz-yBBt7Ao2EM07AAJf0k-6nXDnSLiJhdk9ABhlCreRA).

Our classification of natural and urban sites was based on visual impression during field work. The land cover data in this table largely supports our fieldwork-based natural/urban split. There are a few apparent anomalies. For example, Martin Mere is described as 52% ‘sport and leisure facilities’ – however these facilities are actually a nature reserve with public access. In addition the land cover analysis for West Kirby fails to identify the more rural nature of the ‘discontinuous urban fabric’ (which is connected to a much more open agricultural landscape, with clearly rural habitats separating the site from the large urban conurbation of Liverpool and Birkenhead).

**Figure S1.** Non-linear regression model of the total number of seeds in mallard (A) and Canada Goose (B) samples according to collection date, using a 75% smoothing span. Note the change in scale on the X axis.


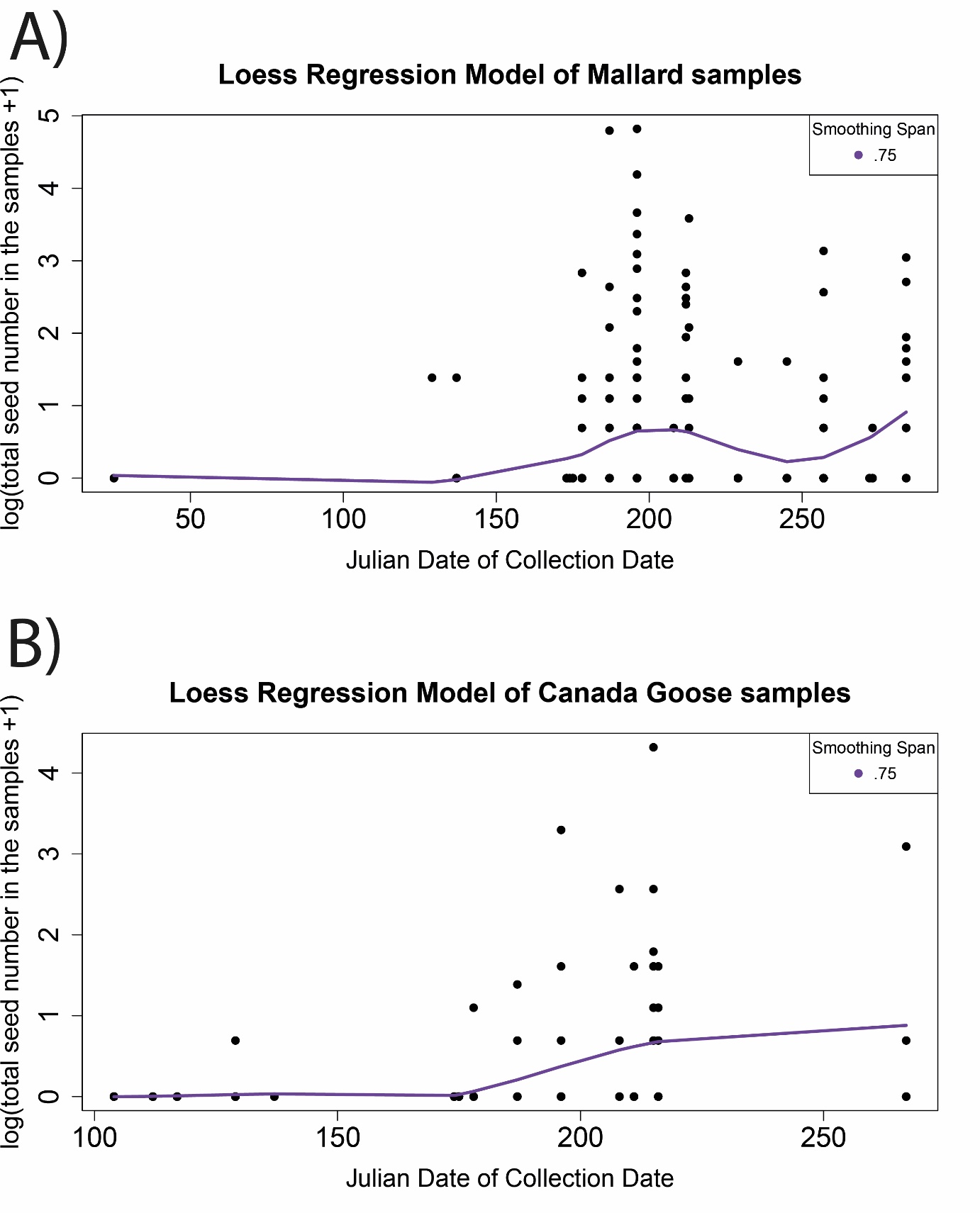


**Figure S2.** Non-linear regression model of the total number of seeds (log-transformed) in samples collected in urban (A) natural habitats (B) according to collection dates, using a 75% smoothing span. Note the change in scale on the X axis.

**
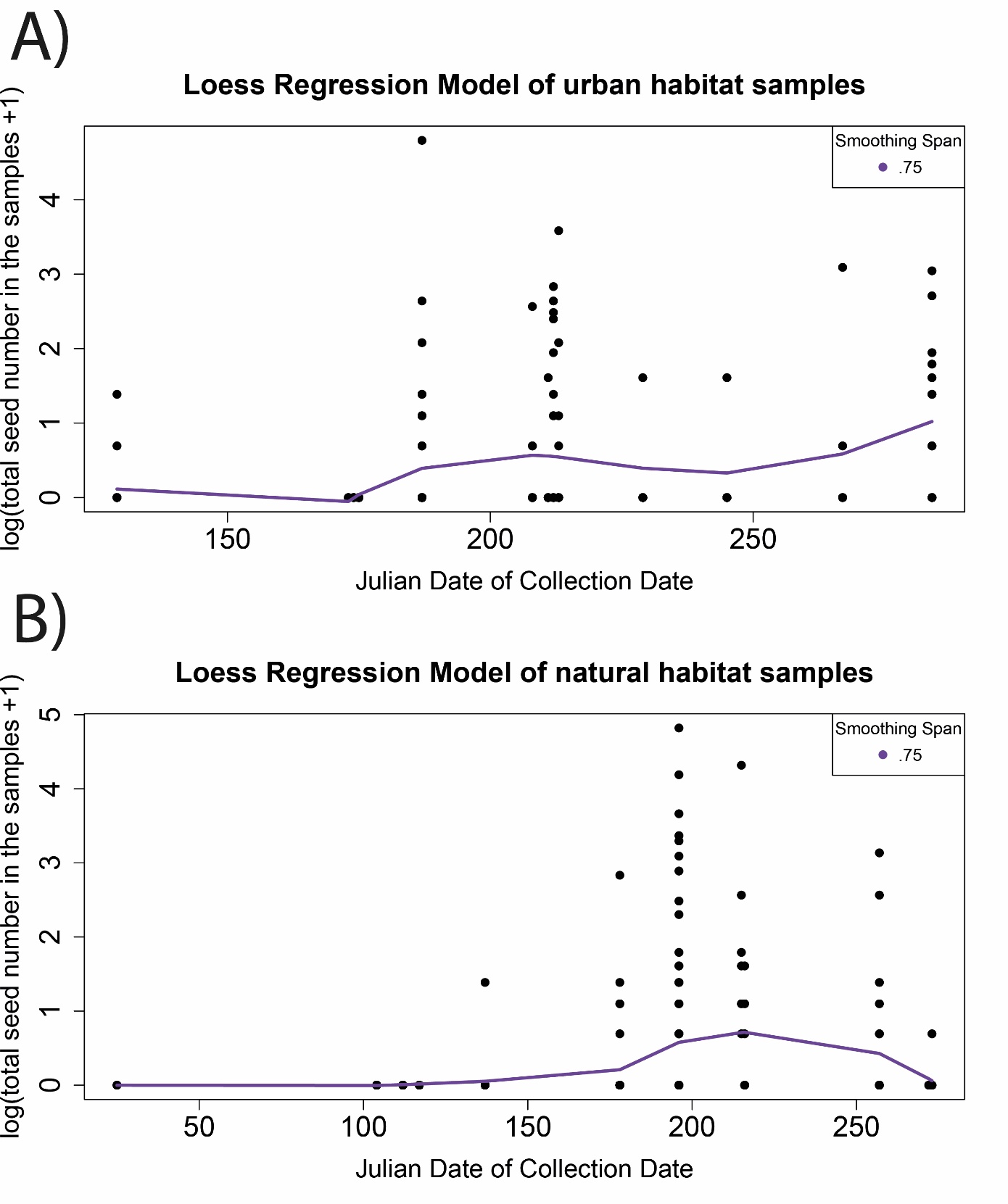
**

**Figure S3.** Rarefaction analysis showing the accumulated number of plant species found in Canada goose and mallard faecal samples according to the number of samples in each habitat type. Error bars represent 95% confidence intervals.


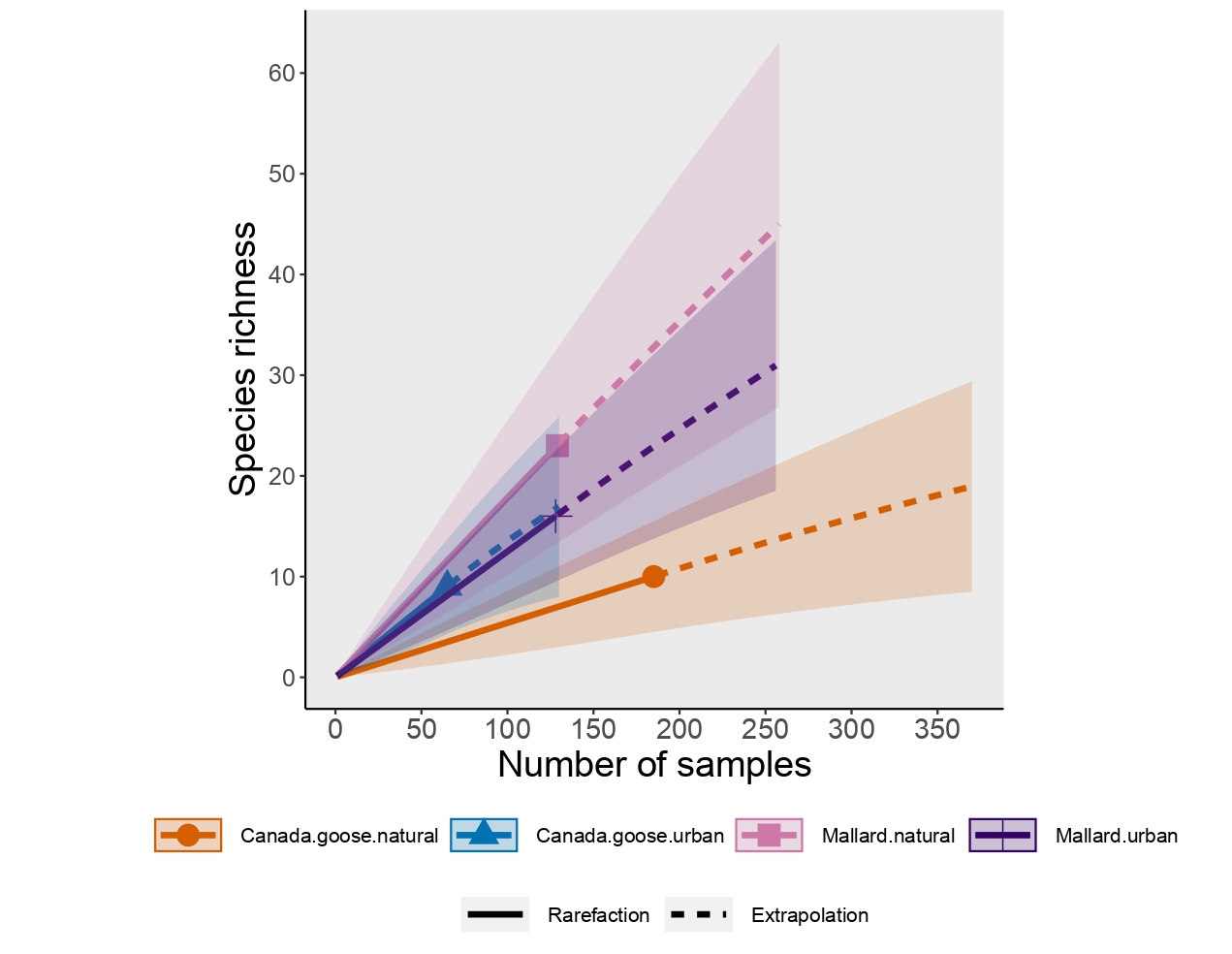


**Figure S4.** Weighted mean + SE of length (A) and width (B) of seeds recovered from samples from different wildfowl. SD and outliers are also shown. For a given sample, the mean values for all seeds present were calculated, prior to calculating the overall distributions shown in the figure..


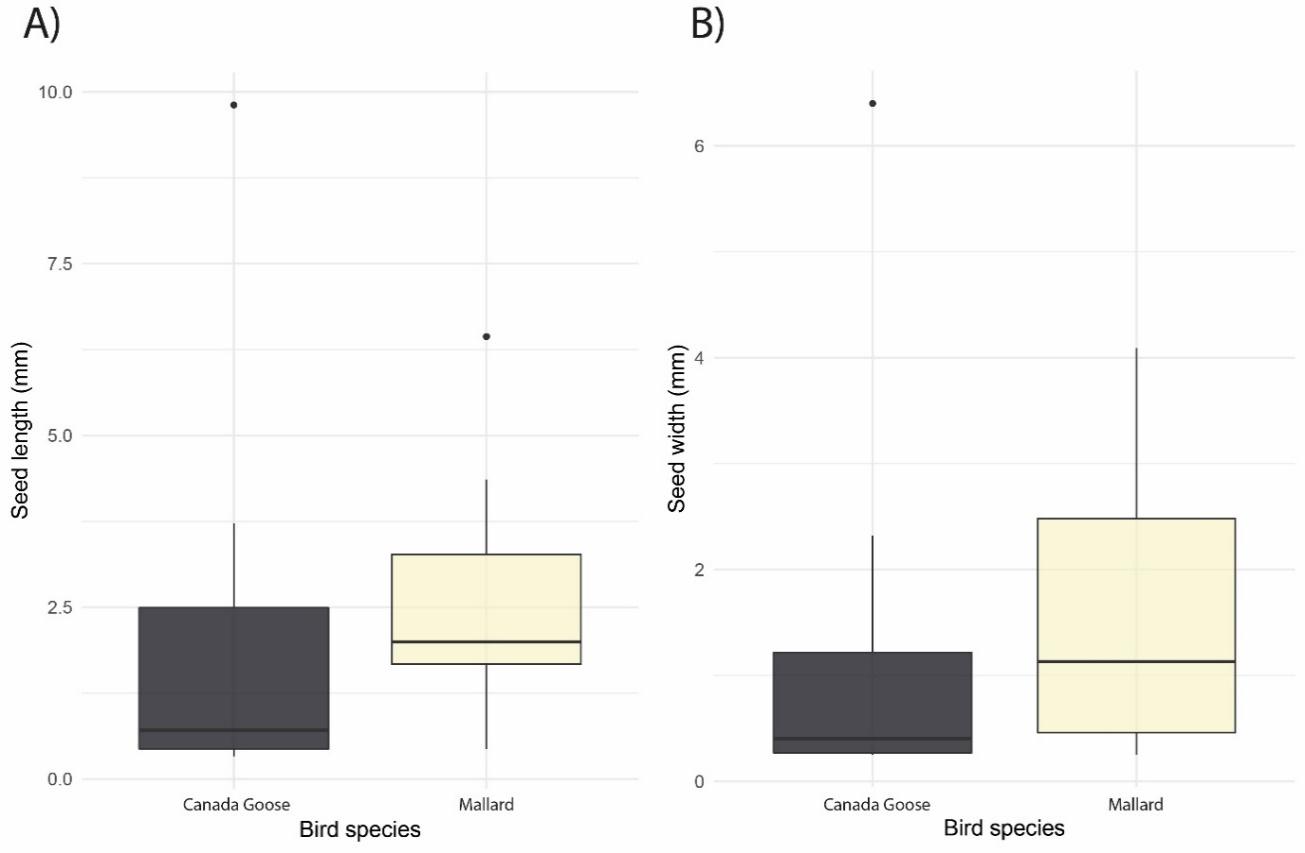

Supplement: Supplementary file 1 — Appendix S1. [file ECE3-13-e10677-s001.docx]
